# Insights Into Patient Variability During Ivacaftor-Lumacaftor Therapy in Cystic Fibrosis

**DOI:** 10.3389/fphar.2021.577263

**Published:** 2021-08-02

**Authors:** Patrick O. Hanafin, Isabelle Sermet-Gaudelus, Matthias Griese, Matthias Kappler, Helmut Ellemunter, Carsten Schwarz, John Wilson, Marsha Tan, Tony Velkov, Gauri G. Rao, Elena K. Schneider-Futschik

**Affiliations:** ^1^ Division of Pharmacotherapy and Experimental Therapeutics, UNC Eshelman School of Pharmacy, The University of North Carolina at Chapel Hill, Chapel Hill, NC, United States; ^2^ Centre Maladie Rare Mucoviscidose, Hôpital Necker-Enfants Malades, Assistance-Publique Hôpitaux de Paris, Paris, France, Institut Necker-Enfants Malades, INSERM U1151, Université Paris Sorbonne, Paris, France; ^3^ Dr. von Hauner Children’s Hospital, University Hospital, LMU, Munich, German Center for Lung Research, München, Germany; ^4^ Department of Child and Adolescent Health, Division of Cardiology, Pulmonology, Allergology and Cystic Fibrosis, Cystic Fibrosis Centre, Medical University of Innsbruck, Innsbruck, Austria; ^5^ Division of Cystic Fibrosis, Department of Pediatric Pneumology, Immunology and Intensive Care, Universitaetsmedizin-Berlin, Berlin, Germany; ^6^ CF Center Westbrandenburg, Campus Potsdam, Berlin, Germany; ^7^ Department of Medicine, Monash University, The Alfred Hospital, Melbourne, VIC, Australia; ^8^ Cystic Fibrosis Service, The Alfred Hospital, Melbourne, VIC, Australia; ^9^ Department of Biochemistry and Pharmacology, School of Biomedical Sciences, Faculty of Medicine, Dentistry and Health Sciences, The University of Melbourne, Parkville, VIC, Australia

**Keywords:** cystic fibrosis, ivacaftor, lumacaftor, drug- drug interactions, cytochrome interactions, pharmacokinetic, pharmacodynamics

## Abstract

**Background:** The advent of cystic fibrosis transmembrane conductance regulator protein (CFTR) modulators like ivacaftor have revolutionised the treatment of cystic fibrosis (CF). However, due to the plethora of variances in disease manifestations in CF, there are inherent challenges in unified responses under CFTR modulator treatment arising from variability in patient outcomes. The pharmacokinetic (PK) data available for ivacaftor-lumacaftor cystic fibrosis (CF) transmembrane conductance regulator (CFTR) modulator drug combination is limited.

**Methods:** Secondary objectives were to identify (1) patient characteristics and (2) the interactions between ivacaftor-lumacaftor responsible for interindividual variability (IIV).

**Results:** Peak plasma concentrations (C_max_) of ivacaftor - lumacaftor were >10 fold lower than expected compared to label information. The one-way ANOVA indicated that the patient site had an effect on C_max_ values of ivacaftor metabolites ivacaftor-M1, ivacaftor-M6, and lumacaftor (*p* < 0.001, *p* < 0.001, and *p* < 0.001, respectively). The Spearman’s rho test indicated that patient weight and age have an effect on the C_max_ of lumacaftor (*p* = 0.003 and *p* < 0.001, respectively) and ivacaftor metabolite M1 (*p* = 0.020 and *p* < 0.001, respectively). Age (*p* < 0.001) was found to effect on C_max_ of ivacaftor M6 and on T_max_ of ivacaftor M1 (*p* = 0.026). A large impact of patient characteristics on the IIV of PK parameters C_max_ and T_max_, was observed among the CF patients.

**Conclusion:** Understanding the many sources of variability can help reduce this individual patient variability and ensure consistent patient outcomes.

## Study summary:

• *What is already known about this subject:* The pharmacokinetic (PK) data available for ivacaftor-lumacaftor cystic fibrosis (CF) transmembrane conductance regulator (CFTR) modulator drug combination is limited.
• *What this study adds:*
  ○ Peak plasma concentrations significantly lower than expected compared to label information.
  ○ A high variability in ivacaftor-lumacaftor pharmacokinetics based on e.g. age and weight was observed.
• *Limitations:* Small patient collective with a number of individual variables.

## Introduction

Cystic fibrosis (CF) is an autosomal recessive genetic disorder that affects chloride transport throughout the epithelial cells of the body, resulting in abnormalities in the respiratory, endocrine, gastrointestinal, and reproductive systems (Condren and Bradshaw, 2013; Tan et al., 2020). The dysfunction of the cystic fibrosis transmembrane conductance regulator (CFTR) channel causes dehydration of mucosal surfaces subsequently increasing viscous mucus that obstructs luminal compartments in lung, pancreas and intestine (Elborn, 2016; Schneider et al., 2017a). The discovery and development of CFTR modulators that directly influence the dysfunctional chloride channel has had a significant impact on CF treatment world-wide (Ghelani and Schneider-Futschik, 2019; Schneider-Futschik, 2019; Allobawi et al., 2020). Ivacaftor-lumacaftor is a fixed-dose tablet containing a corrector (lumacaftor) and potentiator (ivacaftor) of the CFTR (Deeks, 2016). Ivacaftor monotherapy is approved for children 6 more older suffering from gating mutations. While ivacaftor-lumacaftor therapy is approved for children 2 yr and older with a homozygous F508del mutation and for patients 6 yr and older with homozygous F508del mutation or heterozygous and with one residual function mutation.

The ivacaftor-lumacaftor combination is prescribed as a tablet (200 mg lumacaftor and 125 mg ivacaftor, Table 1) dosed every 12 h. Following its oral administration, both ivacaftor and lumacaftor are readily absorbed from the gut; however, they have low solubility in water (<0.05 μg/ml). It has been reported that high fat meals can improve the absorption of both drugs, resulting in increased area under the curve (AUC), and delayed time to reach peak plasma concentration (T_max,_ Table 1) (McColley, 2016). Lumacaftor and ivacaftor exhibit a T_max_ of ∼3–6 and ∼4 h, respectively, (EMA, 2015). The systemic exposure of lumacaftor is approximately 2-fold higher in healthy individuals compared to patients with CF (Fohner et al., 2017). If given alone, the half-life of ivacaftor is 12–14 h, while the half-life of lumacaftor is 26 h (Tables 1 and Supplementary Table 1) and that of the combination is reduced to 9 h (Fohner et al., 2017).

**TABLE 1:** ADME profile of ivacaftor and ivacaftor-lumacaftor standard therapy.

		Orkambi	
	Kalydeco	Lumacaftor	Ivacaftor + lumacaftor
Pharmacokinetics			
Description	Film-coated tablet: 150 mg		200 mg every 12 h lumacaftor with 125 mg every 12 h ivacaftor
Mean ± SD AUC healthy *vs* CF after 150 mg	10,600 *vs* 5,260 ng*hr/ml		198 ± 64.8 lumacaftor and 3.66 ± 2.25 ivacaftor (µg*h/ml)
Mean ± SD C_max_ healthy *vs* CF after 150 mg	768 *vs* 233 ng/ml		25.0 ± 7.96 μg/ml lumacaftor
			0.602 ± 304 μg/ml ivacaftor
Steady state reached	3–5 days with accumulation ratio 2.2–2.9	After 7 days with accumulation ratio of 1.9	7 days (ivacaftor when given with lumacaftor)
Half-life	12–14 h	26 h	9 h (ivacaftor when given with lumacaftor)
			ivacaftor bioavailability is increased 1.53 fold when given with lumacaftor (in healthy)
Absorption			
Increase in exposure	From 25 mg every 12 h to 450 mg every 12 h	From 25 mg every 12 h to 450 mg every 12 h and from 50 to 1,000 mg every 24 h	(ivacaftor) 150 mg every 12 h to 250 mg every 12 h
Increase in exposure	Increased 2–4 fold if given with fat containing food	Increased 2 fold if given with fat containing food	
Median (range) t_max_	4.0 (3.0; 6.0) h in fed state	4.0 (2.0; 9.0) in fed state	(ivacaftor)
Distribution			
Plasma proteins	99% bound to HSA and AGP	99%	
Volume of distribution	275 mg (in healthy and CF) after single dose; 353 ± 122 L after 150 mg every 12 h for 7 days in healthy	86.0 ± 69.8 L (200 mg lumacaftor every 24 h for 28 days)	
Metabolism			
	CYP3A4	Not extensively metabolized	
Excretion			
Elimination in faeces	88% (22% as M1 and 43% as M6)	51% unchanged	
Urinary elimination	6.6% (ivacaftor, M1 and M6)		
Terminal half-life	12 h after single dose in fed state		
Apparent clearance (CL/F)	17.3 (±8.4) L/h in healthy subjects at steady state after 150 mg dose		25.1L ± 40.5% of CL/F of ivacaftor when given with lumacaftor

ADME, absorption, distribution, metabolism and excretion.

As a CYP3A4 substrate ivacaftor undergoes extensive liver metabolism and is, therefore, affected when administered with CYP3A4 inducers such as lumacaftor (Schneider, 2018). Furthermore, based on *in vitro* data lumacaftor and one of the major metabolites of ivacaftor, ivacaftor-M6 are strong inducers of CYP3A4, impacting the PK profile of ivacaftor by potentially reducing ivacaftor concentrations *in vivo* (Schneider et al., 2016; Schneider, 2018). Additionally, ivacaftor-lumacaftor induces several cytochrome P450 enzymes including 3A4, 2B6, 2C9, 2C19, and p-glycoprotein (EMA, 2015; Robertson et al., 2015; Schneider, 2018). Other factors that are known to cause variability in response across patients are the concomitant intake of high fat containing foods as they result in increased absorption of ivacaftor by ∼2.5 to 4-fold.

This paper presents data on the pharmacokinetics (PK) of ivacaftor-lumacaftor. The objectives of this study were to evaluate the PK interactions between ivacaftor and lumacaftor and to evaluate the effects of patient characteristics on their pharmacokinetics. We observed that patient characteristics such as age and weight resulted in large IIV in PK among CF patients.

## Materials and Methods

The study protocol was approved by Monash University Human Research Ethics Committee (project number 0426) and carried out in conformity with the declaration of Helsinki (amended 1986).

### Patients

35 female and 25 male patients from five different treatment centers in Melbourne (*n* = 1), Berlin (*n* = 8), Innsbruck (*n* = 9), Munich (*n* = 17) and Paris (*n* = 25): currently taking ivacaftor-lumacaftor were included in this study after providing written informed consent. All patients experiencing acute infections or exacerbations were excluded. Demographic data collected for these patients included age, gender, and weight. Patients included did not receive any co-medications and were on standard high fat diets.

### Study Design and Drug Administration

This study was a multiple dose, multi-center, open, observational trial reflecting a “real-life” clinical scenario. Patients received ivacaftor-lumacaftor combination administered as an oral tablet twice daily as a fixed dose combination of 125 mg ivacaftor + 200 mg lumacaftor therapy (Supplementary Table 1). The tablet was recommended to be taken with a high fat meal to ensure maximal absorption.

### Blood Sampling

Blood samples (5 ml) were collected into Vacutainer tubes (Becton Dickinson, Rutherford, NJ, United States) containing ethylene-diamine tetra acetic acid as the anticoagulant. Blood samples for measurements of ivacaftor-lumacaftor were taken over the weekly dosing interval immediately before and over a 12-h interval after administration of ivacaftor-lumacaftor. Blood was equilibrated at 20°C for 10 min and then centrifuged (1500 × *g* at 20°C for 10 min) to separate the plasma. The plasma aliquots were stored at −80°C until analysis.

### Drug Analysis

Ivacaftor, its metabolites (ivacaftor-M1 and ivacaftor-M6), and lumacaftor concentrations in plasma were measured simultaneously with high-performance liquid chromatography (HPLC) coupled with mass spectrometry (MS) with u.v. detection after an ion-pair liquid-liquid extraction (Schneider et al., 2016; Schneider et al., 2017b; Reyes-Ortega et al., 2020).

### Assay Data

The 90% confidence limits around the mean assay biases of duplicate quality control samples were determined at low, medium and high concentrations from pooled batches using the LC/MS. The closeness of agreement between a series of measurements obtained from multiple sampling of the same homogeneous sample under the prescribed conditions is expressed as the precision of the analytical procedure. We considered precision at three levels: repeatability, intermediate precision and reproducibility. Herein, the precision of the analytical procedure is expressed as relative standard deviation or coefficient of variation of a series of measurements. The relative standard deviation was calculated by taking the standard deviation of the sample set multiplied by 100% and dividing it by the sample set average. The relative standard deviation is expressed as percent:

%RSD = s/x * 100, where s is the standard deviation and x is the average of three independent measurements. The coefficient of variation (CV) acceptable for both ivacaftor and lumacaftor is a reflection of between-batch precision for peak response data.

### Pharmacokinetic Analysis

PK samples were obtained 4–6 h post administration to characterize C_max_ and T_max_, other samples were around 2.5 and 10 h time post administration. Plasma samples were analyzed as previously described to characterize Ivacaftor, its metabolites M1 and M6 and lumacaftor concentrations (Schneider et al., 2016; Schneider et al., 2017b).

### Statistical Analysis

One-way Analysis of Variance (ANOVA) with Tukey post-hoc test was preformed to evaluate the effect of categorical demographic data (sex, site) on PK parameters (C_max_ and T_max_) of ivacaftor, ivacaftor-M1, ivacaftor-M6, and lumacaftor. Spearman’s Rho non-parametric test was performed to measure the strength of the association between the continuous demographic data (age, weight, and height) and PK parameters (C_max_ and T_max_) of ivacaftor, M1, M6, and lumacaftor.

## Results

### Patients

**FIGURE 1:**
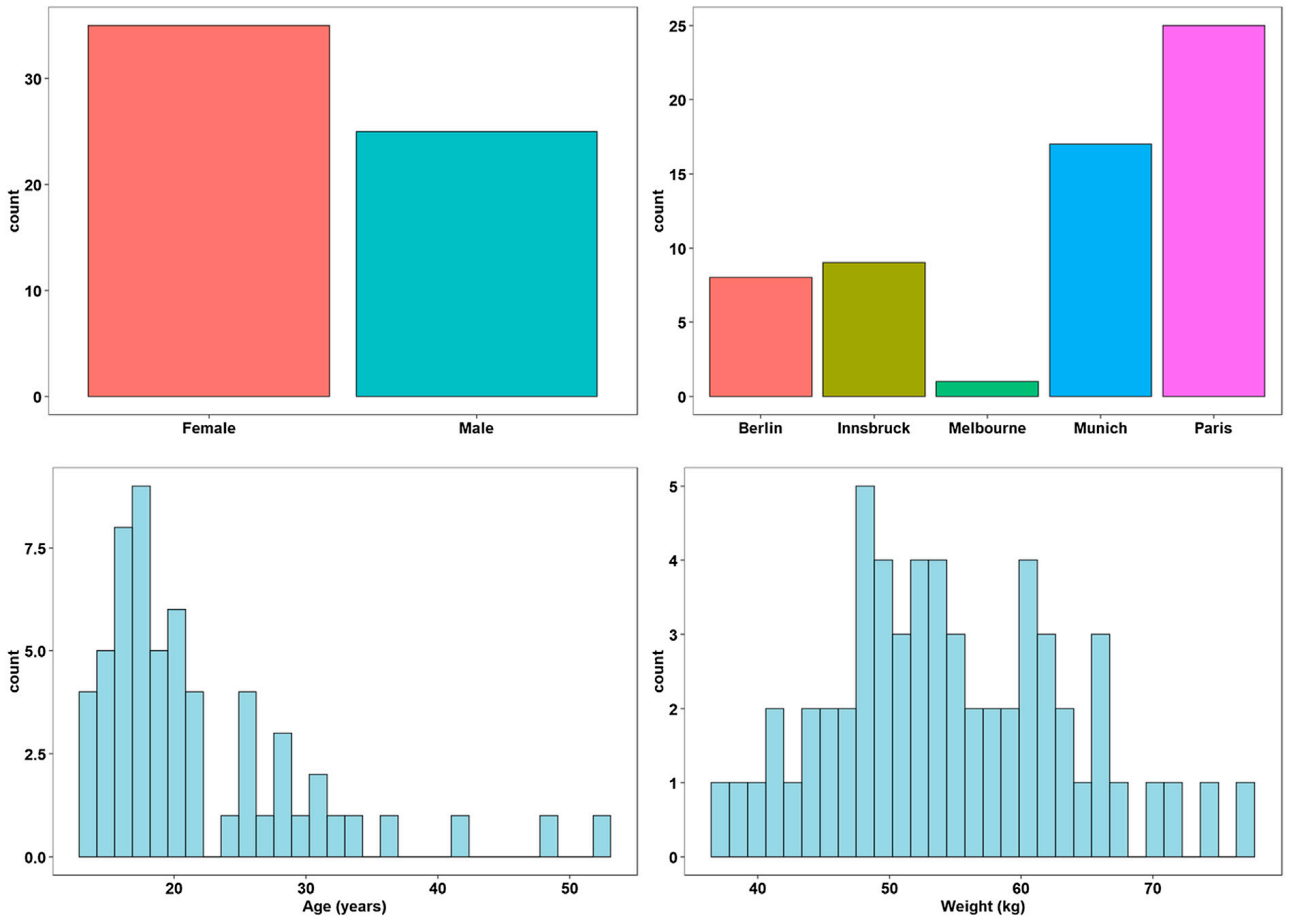
Demographic factors of participants.

A total of sixty Caucasian CF patients (25 male, 35 female) were recruited. The means ± s.d. with (ranges) for patient age and weight were 21.7 ± 7.4 years (13–52 years), 54.3 ± 9.1 kg (37.3–74.8 kg) as reported in Supplementary Table 2. (Figure 1).

### Patient Characteristics

A total of 60 (25 men and 35 women) participated in this study. Patients recruited at each of the sites were: Melbourne (1), Berlin (8), Innsbruck (9), Munich (17) and Paris (25) (Supplementary Table 2). The mean ± s.d. age, weight and height of the subjects were 21.8 ± 8.2 years (range, 13–52 years), 54.5 ± 9.1 kg (range, 37.3–77.2 kg) and 164.8 ± 9.4 cm (range, 148–189.3 cm), respectively (Supplementary Table 2).

### Pharmacokinetic Data

**FIGURE 2:**
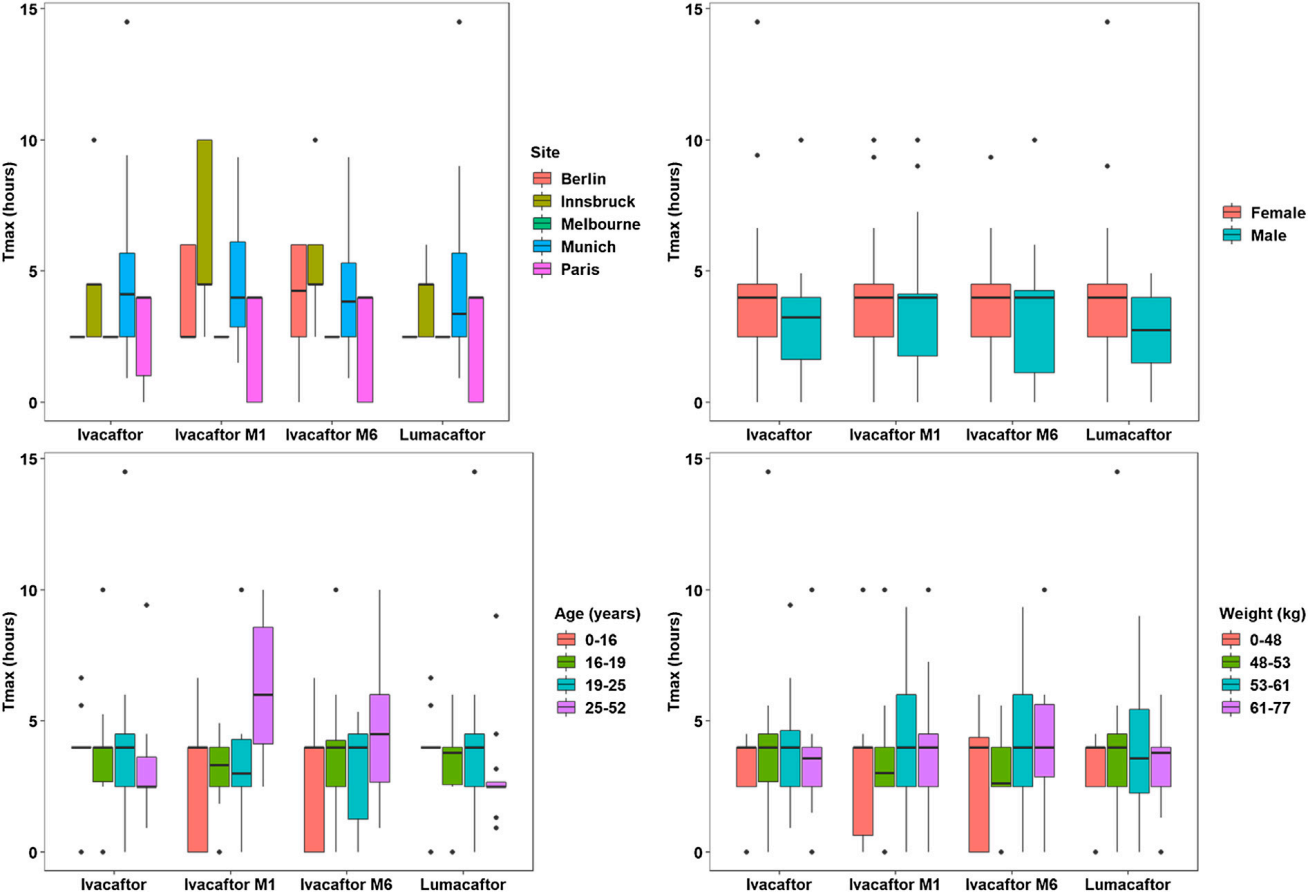
Pharmacokinetic Analysis Plots - T_max_.

A total of 667 pharmacokinetic samples were obtained from the 60 patients (166 ivacaftor, 169 ivacaftor-M1, 163 ivacaftor-M6, and 169 lumacaftor samples). Mean and median plasma PK data for ivacaftor - lumacaftor are summarized in Supplementary Table 3 and illustrated in Figure 2. C_max_ concentrations of ivacaftor - lumacaftor were >10 fold lower compared to the concentrations reported in the drug approval report (EMA, 2015).

### Patient Characteristic Effects on Pharmacokinetics

**FIGURE 3:**
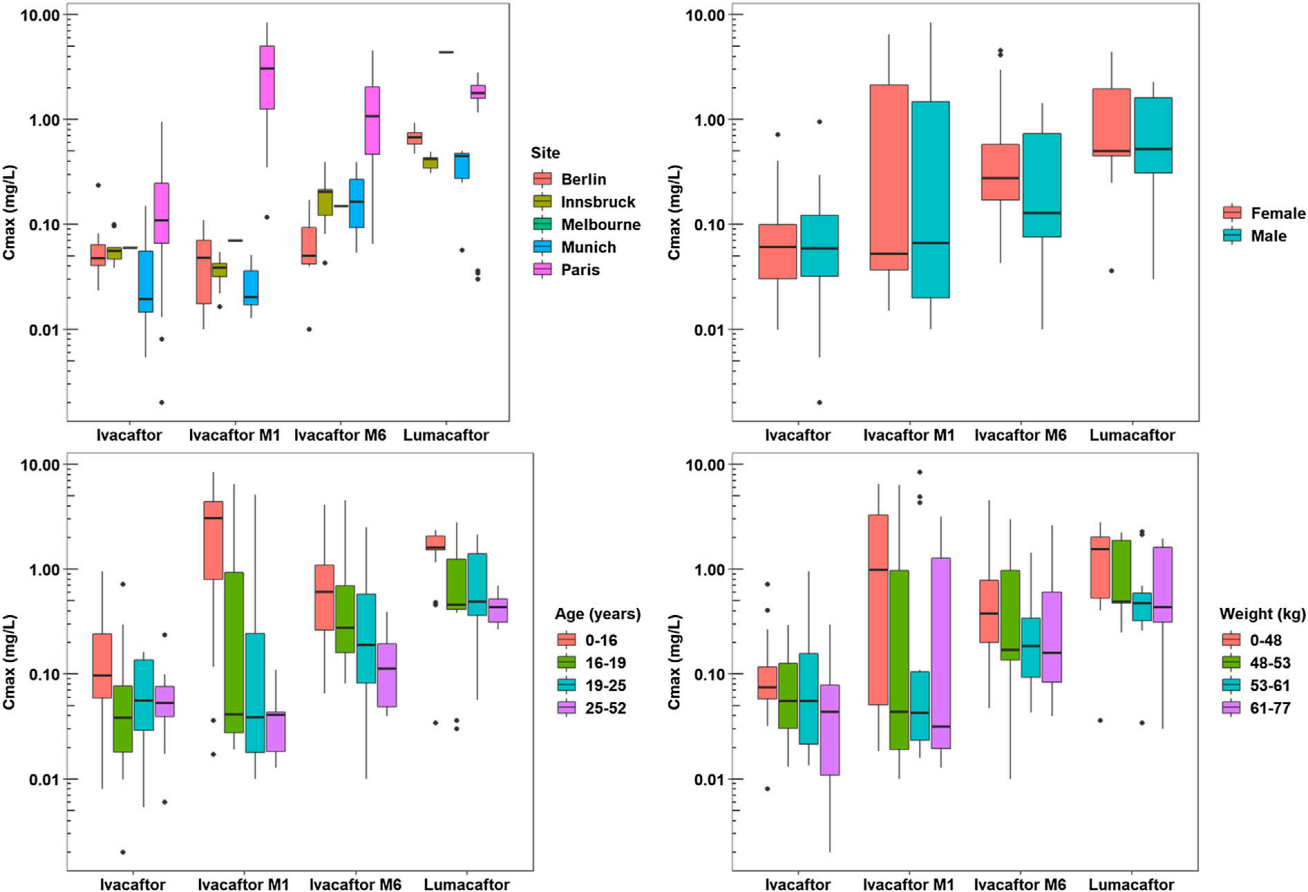
Pharmacokinetic Analysis Plots - C_max_.

The one-way ANOVA indicated that the patient site had a significant effect on the C_max_ values of ivacaftor-M1, ivacaftor-M6, and lumacaftor (*p* < 0.001, *p* < 0.001, and *p* < 0.001, respectively). Additionally, the site has a significant effect on T_max_ for ivacaftor-M1 and ivacaftor-M6 metabolites of ivacaftor (*p* = 0.001 and *p* = 0.004, respectively). The Spearman’s rho test indicated that patient weight and age have a significant effect on the C_max_ of lumacaftor (*p* = 0.003 and *p* < 0.001, respectively) and ivacaftor-M1 (*p* = 0.020 and *p* < 0.001, respectively). Age (*p* < 0.001) was found to have a significant effect on C_max_ of ivacaftor-M6 and on T_max_ of ivacaftor M1 (*p* = 0.026). Patient characteristics had no significant impact on ivacaftor PK. For ivacaftor-M1 site, weight and age had an effect on C_max_, respectively (*p* < 0.001, *p* = 0.02, *p* < 0.001) and for site and age on T_max_ (*p* = 0.001, *p* = 0.026), respectively. For Ivacaftor-M6, both site and age had an impact on C_max_ (*p* < 0.001, *p* < 0.001), respectively and site had an effect on T_max_ (*p* = 0.004). 4) For lumacaftor concentrations site, weight and age, had an effect on C_max_ (*p* < 0.001, *p* = 0.003, *p* < 0.001), respectively (Figures 2, 3).

## Discussion

Drug action is the function of the net absorption, distribution, metabolism, excretion and interactions with target sites which are influenced by genetic factors (Dubovsky, 2015). In CF care, screening for potential drug-drug interactions is of the paramount importance to identify and potentially substitute co-medications that alter drug bioavailability *via* cytochrome P450 (CYP450) induction or inhibition of CYP450. Clinically relevant drug interactions are metabolized by CYP450 enzymes and are divided into families (Condren and Bradshaw, 2013; Tan et al., 2020; Elborn, 2016), sub-families (A-E) and individual gene number. Genetic polymorphisms in a given CYP450 gene result in variations in the enzyme activity leading to poor metabolizers, extensive metabolizers, or ultra-rapid metabolizers. Furthermore, induced cytochrome enzyme activity results in enhanced metabolism of other drug substances, potentially decreasing exposure and reducing therapeutic efficacy. Ivacaftor, a known CYP3A4 substrate, undergoes extensive liver metabolism, and concentrations are likely to be affected when administered concurrently with CYP3A4 inducers (EMA, 2015; VERTEX, 2015). In a recent *in vitro* study, we have reported strong CYP3A4 induction of lumacaftor (Schneider, 2018). These findings together with the data from the EMEA report between lumacaftor and ivacaftor could be at play, wherein the former induces the metabolism of the latter, effectively reducing its effective plasma concentration (EMA, 2015; Schneider, 2018). Ivacaftor is metabolized in the liver by cytochromes CYP3A4 and -A5 with main metabolites produced by oxidation [hydroxymethyl-ivacaftor (ivacaftor-M1) and ivacaftor-carboxylate (ivacaftor-M6)] (Condren and Bradshaw, 2013). Elimination of ivacaftor and metabolites ivacaftor-M1 and ivacaftor-M6 occurs predominantly through the bile (Table 1) (Wainwright, 2014). Lumacaftor is not heavily metabolized with the majority being excreted unchanged in the faeces (EMA, 2015; Sponsor, 2015). Further metabolising enzyme interactions have been reported for ivacaftor including on sensitive substrates of CYP-3A4, -2C8, -2D6 and P-glycoprotein (Robertson et al., 2015). Hence, careful monitoring is recommended when ivacaftor is co-administered with substrates of CYP2C9, CYP3A, and/or P-glycoprotein, particularly drugs with a narrow therapeutic index (Robertson et al., 2015; Paulin and Schneider-Futschik, 2020).

**FIGURE 4:**
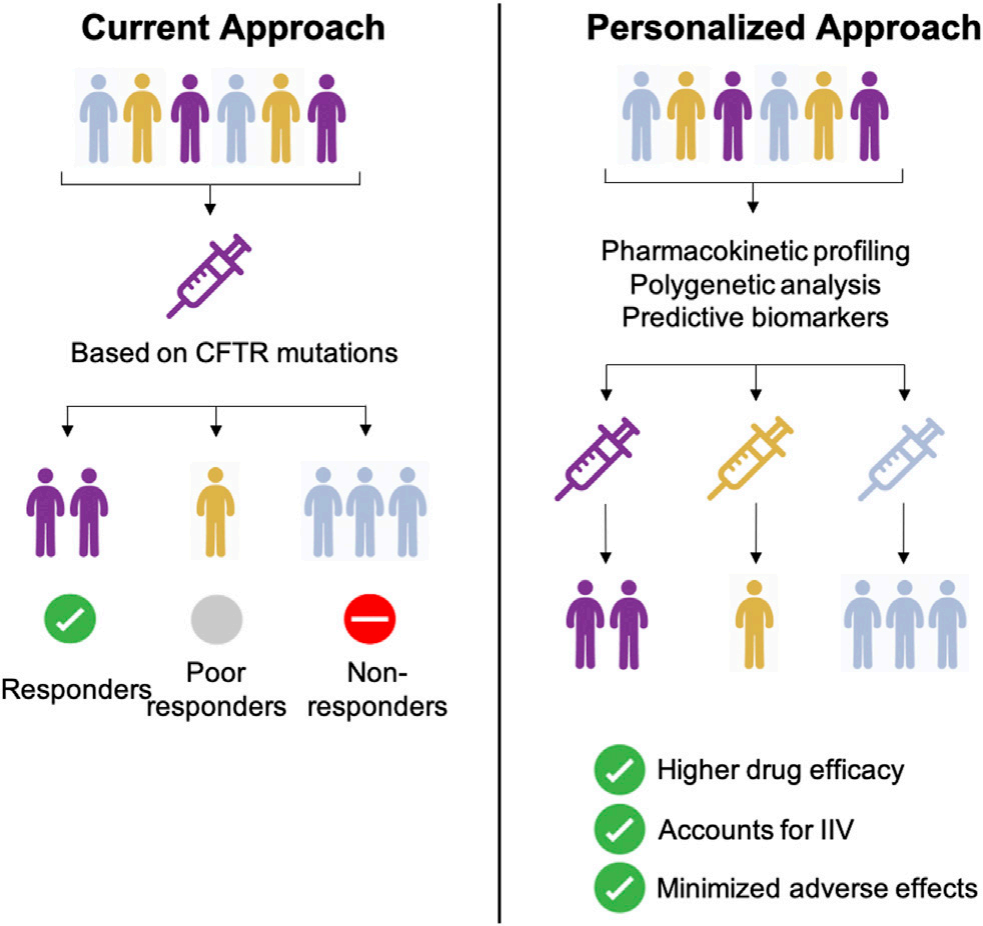
Comparison between current and personalized therapeutic approaches for CF therapy.

Combination therapy of CFTR potentiator and -corrector combinations has progressively transformed quality of life for the majority of patients. Current CFTR combination treatment is selected based on the CFTR mutation e.g. ivacaftor-lumacaftor combination for F508del CFTR mutations. However, clinical relevance of CYP polymorphisms related to dose, effectiveness and/or toxicity are key issues when prescribing other drugs like warfarin, tricyclic antidepressants or proton pump inhibitors. Despite a large number of poor and non-responders under ivacaftor-lumacaftor therapy genomic profiling including cytochrome profiling is not yet considered standard of care (Figure 4, Supplementary Figure 1).

There is an urgent need for well-designed clinical studies aimed at collecting the necessary data to perform population PK analysis in patients undergoing ivacaftor-lumacaftor therapy once they attained steady-state concentrations. The objective of our study was to assess the impact of patient characteristics on the PK of ivacaftor-lumacaftor following multiple doses administered to patients with CF at five different treatment sites. The regimen of 125 mg ivacaftor + 200 mg lumacaftor twice daily is the standard maintenance dose for the treatment of CF (with half-live of 12–13 h for ivacaftor alone; 26 h for lumacaftor alone and 9 h for the combination). Sample collection was performed after patients were on the medication for adequate time to ensure that near steady-state concentrations for both drugs was achieved. We observed that the patient site had a significant effect on the C_max_ values of ivacaftor-M1, ivacaftor-M6, and lumacaftor as well as on T_max_ for the ivacaftor-M1 and ivacaftor-M6. Bioavailability and absorption of ivacaftor are positively correlated with high-fat containing foods (EMA, 2015). A limitation of our study was that the dietary intake of patients at the different sites based on culturally or other choices could not be monitored and could have influenced the absorption of ivacaftor-lumacaftor. Furthermore, we observed that patient weight and age have a significant effect on the C_max_ of lumacaftor and ivacaftor-M1. In particular, age was found to have a significant effect on C_max_ of ivacaftor-M6 and on T_max_ of ivacaftor-M1.

The steady-state exposure of ivacaftor was lower than that seen following one day of therapy and this could be likely due to the CYP3A induction effect of lumacaftor. We observed that C_max_ concentrations of ivacaftor-lumacaftor were >10 fold lower than the values reported in the approval report [9]. Our findings are in line with the respective biomarker study by Masson et al. (2019) (Masson et al., 2019). The authors investigated the biomarker profile and PK/pharmacodynamics (PD) on ivacaftor-lumacaftor in 41 French patients utilizing potential screening tools for predicting PK/PD and patient outcomes utilizing CF biomarkers such as sweat chloride, β-adrenergic peak sweat, lung function (percentage predicted FEV1 [ppFEV1] and residual volume) and CFTR activity. A 5% improvement in FEV1 after 6 months of ivacaftor-lumacaftor therapy was determined as the clinical response threshold. The authors found that only 15 patients had an increase in ppFEV1 of at least 5% (average improvement 13.5% [2.1]; *p* < 0.0001) and were classified as responders; however a majority of patients had a ppFEV1 below 5% and were classified as non-responders (*n* = 21, average change of ∼1% [0.8]; NS). Taken together, reliable *in vivo* biomarkers which predict non-, poor and normal responders for ivacaftor-lumacaftor therapy are urgently required, emphasizing the importance of comprehensive genetic and pharmacogenomic studies in patients using CFTR modulators.

Further patient factors that influence patient outcome in clinical practice ranging from PK/PD parameters to concomitant medications or diets. A decrease in bioavailability or reduction in protein binding from displacement interactions is known to be able to produce decreases in total exposure with no changes in half-life (MacKichan, 1989). In a study looking at human liver microsomes Robertson *et al* (2015) reported the potential of ivacaftor to inhibit p-glycoprotein (pgp) (Robertson et al., 2015). Inhibition of intestinal pgp is known to decrease systemic bioavailable of drugs (Seden et al., 2010; Dubovsky, 2015). As most CF patients receive pancreatic supplementation with meals, fat malabsorption is also a potential factor that could affect the absorption of lipophilic drugs such as ivacaftor.

Based on the complicated PK/PD and drug-drug interaction issues and our data presented in this study we hypothesise whether a personalized approach combining the identification of CFTR mutations with PK/PD and cytochrome identification studies before initiating CFTR modulators. Personalized medicine approaches and personalized biomarker identification have successfully been applied in other disease models including anaplastic lymphoma kinase biomarker for lung cancer (Cutter and Liu, 2012) or pharmacogenomic approaches in multiple sclerosis (Zhou et al., 2019). Carefully constructed PK/PD models together with biomarker identification and pharmacogenomic profiling for CF are becoming more and more indispensable for various reasons: *Firstly*, by anticipating how a subset of patients e.g. CFTR mutation and cytochrome status, might react to CFTR modulator treatment, better clinical trials stratifying the patient subset, can be designed to evaluate patient outcomes. *Secondly*, these models can be used to investigate a number of co-variates including age, weight or gender. An additional difficulty with CF disease is that with more than 2,000 different disease-causing mutations, clinical manifestations vary (pancreatic insufficiency, liver impairment, CF-related diabetes) which can complicate the interpretation of scientific and clinical results (Supplementary Figure 1) (Paulin and Schneider-Futschik, 2020). Factors that can influence patient specific outcomes in CF range from patient specific genetic backgrounds (CFTR mutations, CYP metabolism), inter-patient variables (age, BMI, weight, gender) or others including disease severity and co-morbidities (Supplementary Figure 1). Another unique and non-invasive tool to explore inter-individual pharmacokinetic variability is exhaled breath metabolomics. Nuclear magnetic resonance-based metabolomics of exhaled breath condensate which has successfully been employed to recognize biomarkers of respiratory diseases such as asthma or chronic obstructive pulmonary disease could furthermore contribute to the individual tailoring of treatment for CF (Montuschi et al., 2014; Montuschi et al., 2018). In order to illustrate a personalized medicine approach, clinical and genetic biomarkers need to be identified and PK/PD model that express the dynamic behaviour of the CFTR drug effect need to be developed in patients that are classified as non-/poor and normal responders (Figure 4). In a second step, the relationship between the plasma concentration of a given drug, its corresponding clinical effect and reliable biomarkers need to be identified. When investigating the effect of covariates by stratifying based on current data available taking a personalised approach based on mutation-specific modulator treatment e.g. F508del in combination with determining the cytochrome profile e.g. rapid CYP3A4 metaboliser of each patient is best to be employed. Despite the added cost of these pharmacogenomic tests, unwanted drug-drug interactions due to polypharmacy in the CF population will be minimised. Similarly, the implementation of stem cell and organoid models can pave the way to validate novel surrogate biomarkers of clinical response for CFTR modulators as currently employed in the Netherlands.

Given the genetic and clinical heterogeneity in CF patients developing PK/PD models including comprehensive genetic markers can be utilized as a benchmark to ensure accurate prediction of biomarkers that account for inter-patient variability, higher drug efficacy and minimal adverse effects. To demonstrate the true clinical benefit of genotyping (and not just CFTR mutation identification) for predicting treatment responses for patients under CFTR modulator treatment, it would be necessary to conduct prospective comparisons of different treatments chosen by genotype; which has not been done yet (treatment is chosen based on only the mutation e.g. ivacaftor-lumacaftor for F508del CFTR). Funding sufficiently powered controlled studies of combining genotyping with CFTR mutations and clinical biomarkers is likely to be a challenge.

## Conclusion

In summary, lumacaftor had variable effects on ivacaftor pharmacokinetics including reducing steady-state concentrations. Significant weight and age effects were observed under standard ivacaftor-lumacaftor therapy. Future studies to include both more patients as well as genotyping/PK/PD approaches are warranted to determine if lumacaftor also has variable effects on the PK of ivacaftor.

## Data Availability

The raw data supporting the conclusions of this article will be made available by the authors, without undue reservation.
