# Supplementary material for: Insights Into Patient Variability During Ivacaftor-Lumacaftor Therapy in Cystic Fibrosis
Source: Front Pharmacol. 2021 Aug 2;12:577263. doi: 10.3389/fphar.2021.577263 (PMC8365608; doi:10.3389/fphar.2021.577263)
Supplement: Supplementary file 1 [file DataSheet1.pdf]

Supplementary Information

Insights into the patient variability during ivacaftor-lumacaftor therapy in cystic fibrosis

Patrick O. Hanafin[1], Isabelle Sermet-Gaudelus[2], Matthias Griese[3], Matthias Kappler[3], Helmut Ellemunter[4], Carsten Schwarz[5], John Wilson[6,7], Marsha Tan[8], Tony Velkov[8], Gauri G. Rao[1], Elena K. Schneider-Futschik[8]

Affiliations: [1]Division of Pharmacotherapy and Experimental Therapeutics, UNC Eshelman School of Pharmacy, The University of North Carolina at Chapel Hill, Chapel Hill, NC 27599; [2]Centre Maladie Rare Mucoviscidose, Hôpital Necker-Enfants Malades, Assistance-Publique Hôpitaux de Paris, 149 rue de sèvres, 75015 Paris, France; Institut Necker-Enfants Malades, INSERM U1151, 149 rue de Sèvres, 75015 Paris, France; Université Paris Sorbonne, 75005 Paris, France; [3]Dr. von Hauner Children´s Hospital University Hospital Munich, German Center for Lung Research Lindwurmstr. 4, 80337 München; [4]Department of Child and Adolescent Health, Division of Cardiology, Pulmonology, Allergology and Cystic Fibrosis, Cystic Fibrosis Centre, Medical University of Innsbruck, Innsbruck, Austria; [5]Division of Cystic Fibrosis, Department of Pediatric Pneumology, Immunology and Intensive Care, Universitaetsmedizin-Berlin, Berlin, Germany; [6] Dept of Medicine, Monash University, The Alfred Hospital, Melbourne, Australia; [7]Cystic Fibrosis Service, The Alfred Hospital, Melbourne, Australia; [8]Department of Pharmacology & Therapeutics, School of Biomedical Sciences, Faculty of Medicine, Dentistry and Health Sciences, The University of Melbourne, Parkville, VIC, 3010, Australia;

Correspondence: elena.schneider@unimelb.edu.au

Supplementary Table 1. Parameters from 125 mg ivacaftor + 200 mg lumacaftor combination label information

	Ivacaftor	Lumacaftor
Initial Parameters		
T_{max}	4h	4h
CL/F	25.1 L/h	2.38 L/h
V/F		86L
Half-life	9h	26h
Dose	250 mg	400 mg
AUC	3.66 ug/mL*h	198 ug/mL*h
C_{max}	0.602 ug/mL	25 ug/mL
Calculated Parameters		
k_{el}	0.077 1/hr	0.0277 1/h
k_a	0.583 1/hr	0.897 1/h

T_{max} = amount of time that a drug is present at the maximum concentration; CL/F = oral clearance; V/F = Drug volume of distribution; AUC = Area under the curve; C_{max} = amount of drug is present at the maximum concentration; k_{el} = elimination rate constant; k_a = absorption rate constant

Supplementary Table 2. Demographics of CF patients

	**Age (y)**	**Weight (kg)**	**Height (cm)**
Max	52	77.2	189.3
3rd Quartile	25	61.1	171.0
Median	19	53	164.0
Mean	21.8	54.5	164.8
1st Quartile	16	48	158.8
Min	13	37.3	144.5
Missing	1	1	1
STD	8.17	9.08	9.65

Footnote: The total number of patients N = 60 (Site: Melbourne: 1, Berlin: 8, Innsbruck: 9, Munich: 17, Paris: 25) including 35 female and 25 male patients.

Supplementary Table 3. Pharmacokinetic Analysis - T_{max} and C_{max}

	Ivacaftor	Ivacaftor-M1	Ivacaftor-M6	Lumacaftor
		T_{max} (hours)		
Max	14.5	10	10	14.5
3rd Quartile	4	4.5	4.5	4
Median	4	4	4	4
Mean	3.58	3.88	3.486	3.39
1st Quartile	2.5	2.5	2.5	2.5
Min	0	0	0	0
STD	9.36	10.5	11.3	9.63
Missing	1	2	3	1
		C_{max} (mg/L)		
Max	0.952	8.42	4.56	4.42
3rd Quartile	0.100	1.35	0.61	1.70
Median	0.059	0.044	0.206	0.503
Mean	0.107	1.27	0.627	1.06
1st Quartile	0.024	0.020	0.085	0.415
Min	0	0	0	0.030
STD	0.160	2.14	0.988	0.899

Supplementary Figure 1.

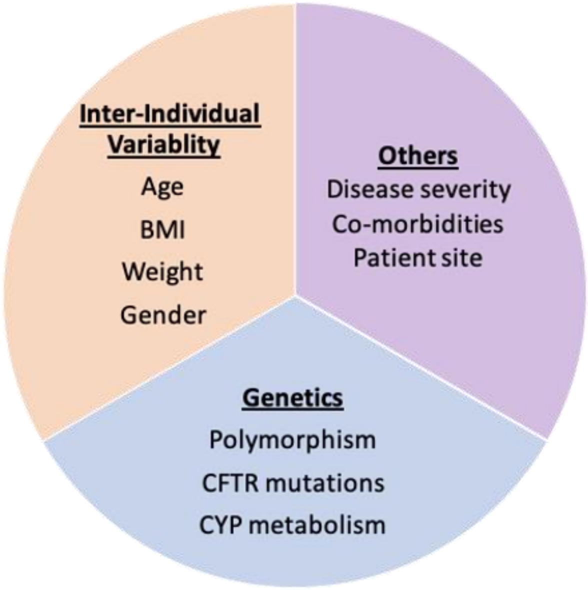

Supplementary Figure 1. Overview of genetic, inter-individual and other factors contributing to poor responses for CF modulator therapy
